# Abnormal meiosis in an intersectional allotriploid of *Populus* L. and segregation of ploidy levels in 2x × 3x progeny

**DOI:** 10.1371/journal.pone.0181767

**Published:** 2017-07-21

**Authors:** Jun Wang, Beibei Huo, Wanting Liu, Daili Li, Ling Liao

**Affiliations:** 1 Beijing Advanced Innovation Centre for Tree Breeding by Molecular Design, Beijing Forestry University, Beijing, People’s Republic of China; 2 National Engineering Laboratory in Tree Breeding, Beijing Forestry University, Beijing, People’s Republic of China; 3 Key Laboratory of Genetics and Breeding in Forest Trees and Ornamental Plants, MOE, Beijing Forestry University, Beijing, People’s Republic of China; 4 College of Biological Sciences and Technology, Beijing Forestry University, Beijing, People’s Republic of China; Institut de Genetique et Developpement de Rennes, FRANCE

## Abstract

Triploid plants are usually highly aborted owing to unbalanced meiotic chromosome segregation, but limited viable gametes can participate in the transition to different ploidy levels. In this study, numerous meiotic abnormalities were found with high frequency in an intersectional allotriploid poplar (*Populus alba* × *P*. *berolinensis* ‘Yinzhong’), including univalents, precocious chromosome migration, lagging chromosomes, chromosome bridges, micronuclei, and precocious cytokinesis, indicating high genetic imbalance in this allotriploid. Some micronuclei trigger mini-spindle formation in metaphase II and participate in cytokinesis to form polyads with microcytes. Unbalanced chromosome segregation and chromosome elimination resulted in the formation of microspores with aneuploid chromosome sets. Fusion of sister nuclei occurs in microsporocytes with precocious cytokinesis, which could form second meiotic division restitution (SDR)-type gametes. However, SDR-type gametes likely contain incomplete chromosome sets due to unbalanced segregation of homologous chromosomes during the first meiotic division in triploids. Misorientation of spindles during the second meiotic division, such as fused and tripolar spindles with low frequency, could result in the formation of first meiotic division restitution (FDR)-type unreduced gametes, which most likely contain three complete chromosome sets. Although ‘Yinzhong’ yields 88.7% stainable pollen grains with wide diameter variation from 23.9 to 61.3 μm, the pollen viability is poor (2.78% ± 0.38). A cross of ‘Yinzhong’ pollen with a diploid female clone produced progeny with extensive segregation of ploidy levels, including 29 diploids, 18 triploids, 4 tetraploids, and 48 aneuploids, suggesting the formation of viable aneuploidy and unreduced pollen in ‘Yinzhong’. Individuals with different chromosome compositions are potential to analyze chromosomal function and to integrate the chromosomal dosage variation into breeding programs of *Populus*.

## Introduction

Polyploidization is an important driving force in plant speciation and evolution [1,2]. During the evolution process of plants, crosses between different ploidy levels contribute to chromosomal introgression and genetic diversity [3–5]. Interploidy hybridization is also a useful pathway for the creation of new germplasms and crops varieties [6,7]. However, polyploids with odd numbers of chromosome sets, especially triploids, are usually unstable, which can either be sterile to be aborted in the evolution process due to their reproductive barrier, or contribute to production of polyploid gametes, depending on the species [8–10].

Polyploids with odd ploidy levels have difficulty carrying out regular meiosis owing to their genetic imbalance. Irregular chromosome pairing and unequal meiotic division commonly occur, resulting in unbalanced chromosome segregation and chromosome elimination [11]. Therefore, each spore after asymmetrical cytokinesis contains incomplete chromosome sets, i.e., aneuploidy, and the aneuploid unbalanced chromosomal composition causes high levels of sterility in gametes. Nevertheless, some aneuploid gametes can take part in fertilization to produce aneuploid progeny [12–16]. Additionally, meiotic nuclear division restitution in odd polyploids can also produce unreduced viable gametes, although the production of unreduced gametes is low frequency [17]. The production of viable aneuploidy and unreduced gametes suggests that polyploids with odd ploidy levels also have value in acting as a bridge for the induction of chromosomal number variation [17–19].

Aneuploids are particularly valuable for chromosome engineering breeding and cytogenetic research. Monosomic, nullisomic, trisomic and tetrasomic wheat lines have all been established to analyze chromosomal function and evolutionary relationships and to locate particular genes [20]. In *Arabidopsis thaliana* (L.) heynh., triploids have produced a group of aneuploids by self-pollination [21], and phenotypic variation is strongly associated with chromosome composition and dosage variation in *A*. *thaliana* aneuploids [22]. Chromosome manipulation based on aneuploids has attracted increasing attention for improving efficiency in plant breeding.

Poplars are dioecious, beding widely cultivated across the Northern Hemisphere as an important source of fiber, biomass and lumber [23]. They also have been considered a model tree for molecular biological and genetic research in woody plants, owing to the relatively small genome size and short reproductive cycle [24]. Utilization of chromosome number variation is an important approach in *Populus* breeding programs, and many allotriploid poplar cultivars, including triploid *P*. *tomentosa* Carr., *P*. *alba* L. × *P*. *berolinensis* Dippel. ‘Yinzhong’, *P*. *tremula* L. × *P*. *tremuloides* Michx. ‘Astria’, *P*. × *euramericana* (Dode) Guiner CL. ‘Zhonglin-46’, *P*. × *canadensis* Moench cv. ‘Sacrau 79’, and *P*. × *euramericana* (Dode) Guiner cv. ‘Wuhei-1’ [25–28], have been widely used in plantations owing to their favorable growth performance and pulpwood characteristics [29,30]. A series of methods for polyploid induction have been developed to increase the induction efficiency and heterozygosity of polyploid progeny [31–37]. However, chromosomal function and the relationship between chromosome composition and commercial phenotypic variation are poorly characterized in *Populus* owing to the lack of aneuploid germplasms.

*P*. *alba* × *P*. *berolinensis* ‘Yinzhong’ is an artificially synthesized male allotriploid hybrid. The parents *P*. *alba* and *P*. *berolinensis* are both diploid, belonging to two different sections (sect. Populus and sect. Aigeiros, respectively) within the genus *Populus* [38]. It is speculated that ‘Yinzhong’ is derived from the union between a normal gamete and a spontaneous unreduced gamete from one of the parents [26]. Generally, the sect. Populus is reproductively isolated from other sections [39]. Internal transcribed spacer (ITS) sequence analysis showed that sect. Aigeiros and sect. Populus had different evolution ratios [40], suggesting relatively large genetic divergence between the two sections. In this study, we analyzed meiotic abnormalities and pollen variation of the intersectional allotriploid ‘Yinzhong’. A cross between the triploid and a diploid female clone was conducted to produce progeny with ploidy level segregation, aiming to further analyze chromosomal function in *Populus* and integrate the potential for chromosomal dosage variation into *Populus* breeding programs.

## Materials and methods

### Plant materials

Floral branches of allotriploid ‘Yinzhong’ were collected from a plantation in Tongliao City (Inner Mongolia Autonomous Region, People’s Republic of China). The plantation was established by the Forestry Research Institute of Tongliao City, the Inner Mongolia Autonomous Region, People’s Republic of China. We got the permission from the institute to collect the branches for research. For sexual hybridization, a diploid female clone of *P*. *tomentosa* Carr. × *P*. *bolleana* Lauche, TB03 (2n = 2x = 38), collected from the campus of China Agricultural University, was used as the female parent. TB03 has usually been used as the female parent in test crosses of section Populus, because it has good fertility and no spontaneous unreduced egg production was found [41,42]. Floral branches of ‘Yinzhong’ and TB03 were cultured in water in a greenhouse (10–20°C) to force floral development.

### Cytological observations of microsporogenesis

Floral buds of ‘Yinzhong’ undergoing meiotic development were collected and immediately fixed in an ethanol-acetic acid fixative solution (3:1 v/v) at 4°C for 24 h, and then they were stored in 70% ethanol until analysis.

Microsporocytes were pressed out of anthers and stained in 1% aceto-carmine on a microscope slide. To increase chromosome stainability, preparations were heated slightly over a flame before observation. All preparations were observed using a microscope (BX51: Olympus), and photos were taken with an attached video camera (DP70: Olympus). Meiotic pairing configurations were recorded based on 21 early metaphase I cells. The frequency of precocious chromosome migration in metaphase I, laggard chromosomes or bridge formation in anaphase I, the presence of micronuclei in telophase I and II, and the number of parallel, fused and tripolar spindles in anaphase II were analyzed together with the variation of sporad types. The meiotic index was calculated as the fratio of the number of normal sporads to the total number of sporads analyzed per hybrid multiplied by 100 [43].

### Indirect immunofluorescence of microtubules

Indirect immunofluorescence testing was used to analyze the change in the microtubule cytoskeleton according to Wang et al. [44,45]. Anthers were fixed in 4% paraformaldehyde in PEM buffer (50 mM Pipes, 5 mM EGTA, 2 mM MgSO_4_, pH 6.9) for 45 min. After three 5-min rinses in PEM buffer, the anthers were treated with 10% dimethylsulfoxide (DMSO) and 1% Triton X-100 for 15 min each. The anthers were then rinsed in PEM buffer, followed by three 5-min rinses in PBS buffer (137 mM NaCl, 2.7 mM KCl, 7 mM Na_2_HPO_4_, 1.5 mM KH_2_PO_4_, pH 7.3). Subsequently, microsporocytes were squeezed out from the anthers and transferred to a slide coated with 0.1% poly-L-lysine (Sigma-P1274: Sigma-Aldrich, St Louis, MO, USA). The cells were incubated in a monoclonal anti-α-tubulin antibody (Sigma-T9026: Sigma-Aldrich) diluted 1:100 with the PBS buffer for 2 h at 37°C in a moist chamber. Following further washing with the PBS buffer, the microsporocytes were incubated in FITC-conjugated antimouse IgG (Sigma-F0257: Sigma-Aldrich) diluted 1:100 with the PBS buffer for 2 h at 37°C in a dark chamber. After a final wash in the PBS buffer, the microsporocytes were mounted using mounting medium with 4′, 6-diamidino-2-phenylindole (DAPI; Vectashield® H-1200: Vector Laboratories, Inc., Burlingame, CA, USA). The preparations were observed and photographed with a confocal laser scanning microscope (TCS-SP5: Leica, Solms, Germany).

### Analysis of pollen size and viability

A pollen sample was collected from freshly dehiscent anthers on male catkins. The fresh pollen sample was mounted immediately in a drop of 1% aceto-carmine on a microscope slide. Empty and shrunken pollen grains were scored. The diameter of 1,500 stained spherical pollen grains was measured using an ocular micrometer to establish a histogram of the frequency distribution.

The fresh pollen samples were germinated on agar medium containing 15 g L^−1^ agar, 120 g L^−1^ sucrose, 60 mg L^−1^ boric acid and 80 mg L^−1^ calcium nitrate to test its viability. After 24 h of culturing, the germination rates were recorded based on five replications.

### Crossing experiment

When catkins of female parent TB03 acquired stigma receptivity, they were pollinated with fresh ‘Yinzhong’ pollen in a greenhouse. After the catkins matured, seeds were collected and germinated in sterile soil, and the seedlings were transplanted to the field when they reached approximately 30 cm in height.

### Analysis of ploidy levels

Determination of the ploidy level of ‘Yinzhong’ was conducted by somatic chromosome counting. Stem tips were removed from seedlings and pretreated with a saturated solution of paradichlorobenzene for 4 h at 25°C. The materials were then fixed in fresh Farmer’s solution (ethanol/acetic acid, 3:1) for 24 h at 4°C and then hydrolyzed in 38% HCl/ethanol (1:1) for 25 min at room temperature. After washing in distilled water three times for 15 min, the hydrolyzed materials were squashed in a carbol fuchsin solution on a microscope slide. The preparation was observed using an Olympus BX51 microscope.

For the progeny, when seedlings had more than three leaves, a young leaf was used to analyze the ploidy level by flow cytometry. Flow cytometric analysis was conducted according to Galbraith et al. [46]. Briefly, young leaves of the seedlings were chopped in modified Galbraith’s buffer (45 mM MgCl_2_·6H_2_O, 20 mM MOPS, 30 mM sodium citrate, 0.5% Triton X-100, 1% PVP-10, pH 7.0) using a sharp razor blade on ice. Subsequently, the nuclear suspension was filtered through a 40-μm nylon mesh to remove large debris. Nuclei were stained with 50 μg mL^−1^ propidium iodide with RNase at 50 μg mL^−1^. After incubation on ice for 30 min in the dark, samples were analyzed with a BD FACSCalibur flow cytometer. TB03 and ‘Yinzhong’ were used as diploid and triploid criteria, respectively. The somatic chromosome numbers of some seedlings were also checked using the above method.

## Results

### Chromosome counting of triploid ‘Yinzhong’

**Fig 1:**
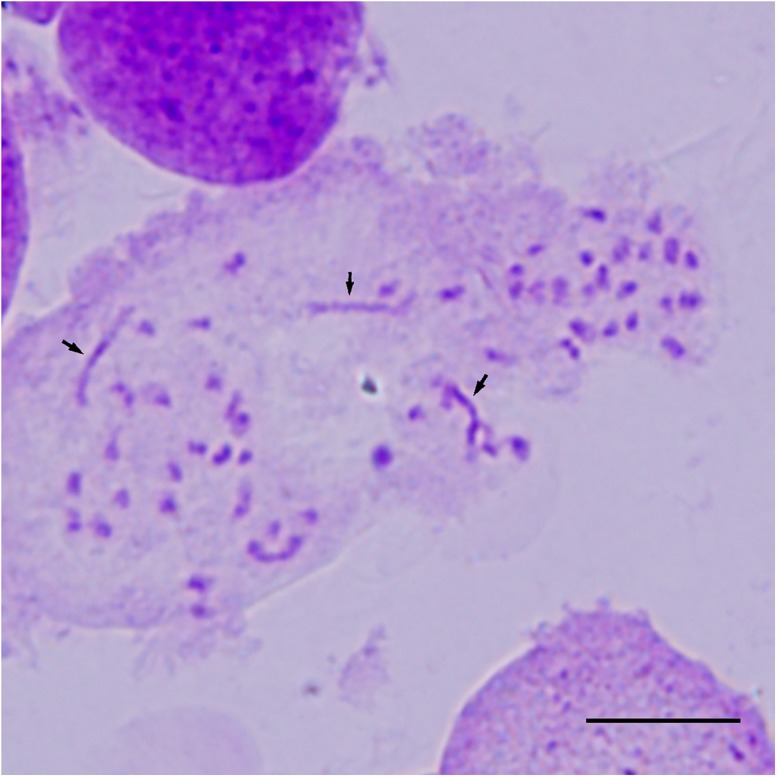
Somatic chromosome counting (2n = 3x = 57) of *P*. *alba* × *P*. *berolinensis* ‘Yinzhong’. Arrows indicate the chromosome I. Bar is equal to 10 μm.

Although ‘Yinzhong’ was previously determined to be a triploid by Chen et al. [26], the ploidy level of the male clone used in this study was detected to avoid errors. Chromosome counting showed that ‘Yinzhong’ had 57 chromosomes with 3 large ones corresponding to chromosome I of *Populus* (Fig 1); this indicates that the male clone is a triploid.

### Abnormal chromosome behaviors during microsporogenesis

**Fig 2:**
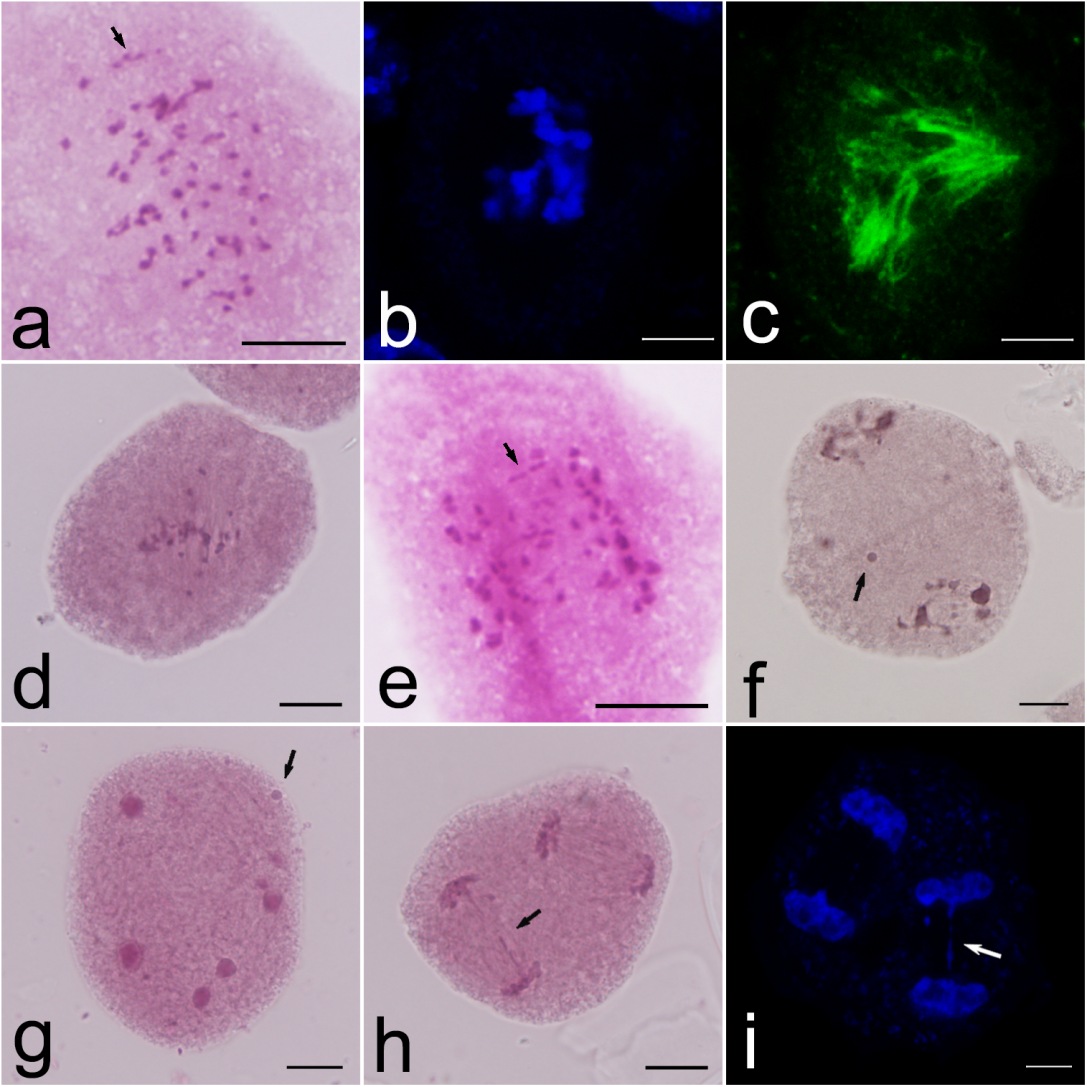
Meiotic abnormalities of the allotriploid *P*. *alba* × *P*. *berolinensis* ‘Yinzhong’. a. Early metaphase I with several univalents and trivalents (arrows); b–c. Metaphase I with a multipolar spindle showing chromosome arrangement (b) and microtubule distribution (c); d. Metaphase I with precociously migrated chromosomes; e. Anaphase I with lagging chromosomes (arrow); f. Telophase I with micronucleus (arrow); g. Telophase II with micronucleus (arrow); h. Anaphase II with chromosome bridge (arrow); i. DAPI stained Telophase II showing chromosome bridge (arrow). a, d–h Bars are equal to 10 μm; b, c, i Bars are equal to 5 μm.

Chromosome pairing was examined at early metaphase I, showing the existence of univalents (I), bivalents (II), and trivalents (III) (Table 1, Fig 2A). The average meiotic configuration was 15.2I + 14.0II + 4.6III. The univalents ranged from 6 to 30, indicating partial failure of uniparental chromosomal pairing and high genomic heterozygosity of ‘Yinzhong’. The trivalents ranged from 1 to 7, suggesting some degree of homology between the two parents of ‘Yinzhong’, although the parents belong to two different sections.

**Table 1:** Meiotic pairing configuration of the allotriploid *P*. *alba* × *P*. *berolinensis* ‘Yinzhong’.

Clone	Chromosome number	No. of early metaphase I cells	Pairing configuration		
			Univalents	Bivalents	Trivalents
Yinzhong	57	21	15.2 (6–30)	14.0 (10–18)	4.6 (1–7)

A large number of meiotic abnormalities were recorded in this allotriploid ‘Yinzhong’ (Table 2), showing unbalanced chromosome segregation. At diakinesis, several univalents and multivalents were found (Fig 2A), suggesting aberrant synapsis in this allotriploid. At metaphase I, abnormal chromosome disposition was observed (Fig 2B), and as a result, the spindle formed an abnormal structure (Fig 2C). Moreover, some chromosomes migrated precociously to the poles in almost all cells (Fig 2D). In anaphase I, lagging chromosomes (Fig 2E) and chromosome bridges were found with frequencies of 59.07% and 6.56%, respectively. Some lagging chromosomes could form micronuclei at telophase I (Fig 2F) with a frequency of 68.87%. During the second meiotic division, micronuclei located in the cytoplasm still were observed (Fig 2G). In anaphase II and telophase II, lagging chromosomes and chromosome bridges were also found (Fig 2H and 2I).

**Table 2:** Meiotic abnormalities in the allotriploid *P*. *alba* × *P*. *berolinensis* ‘Yinzhong’.

Phase	No. of analyzed cells	No. of abnormal cells (%)	Abnormalities	No. of each abnormality
Metaphase I	1,026	1,003 (97.76)	Precocious chromosome migration	1,003
Anaphase I	259	162 (62.55)	Lagging chromosomes	153
			Chromosome bridge	17
Telophase I	379	261 (68.87)	Micronuclei	261
Prophase II	233	201 (86.27)	Micronuclei	174
			Precocious cytokinesis	49
Anaphase II	468	209 (44.66)	Parallel spindles	5
			Fused spindles	11
			Tripolar spindles	8
			Precocious cytokinesis	185
Telophase II	590	468 (79.32)	Micronuclei	328
			Precocious cytokinesis	219
Meiotic product	1,936	263 (13.58)	Dyad	38
			Triad	71
			Polyad	154
Meiotic index		86.42		

### Misorientation of spindles and aberrant cytokinesis

**Fig 3:**
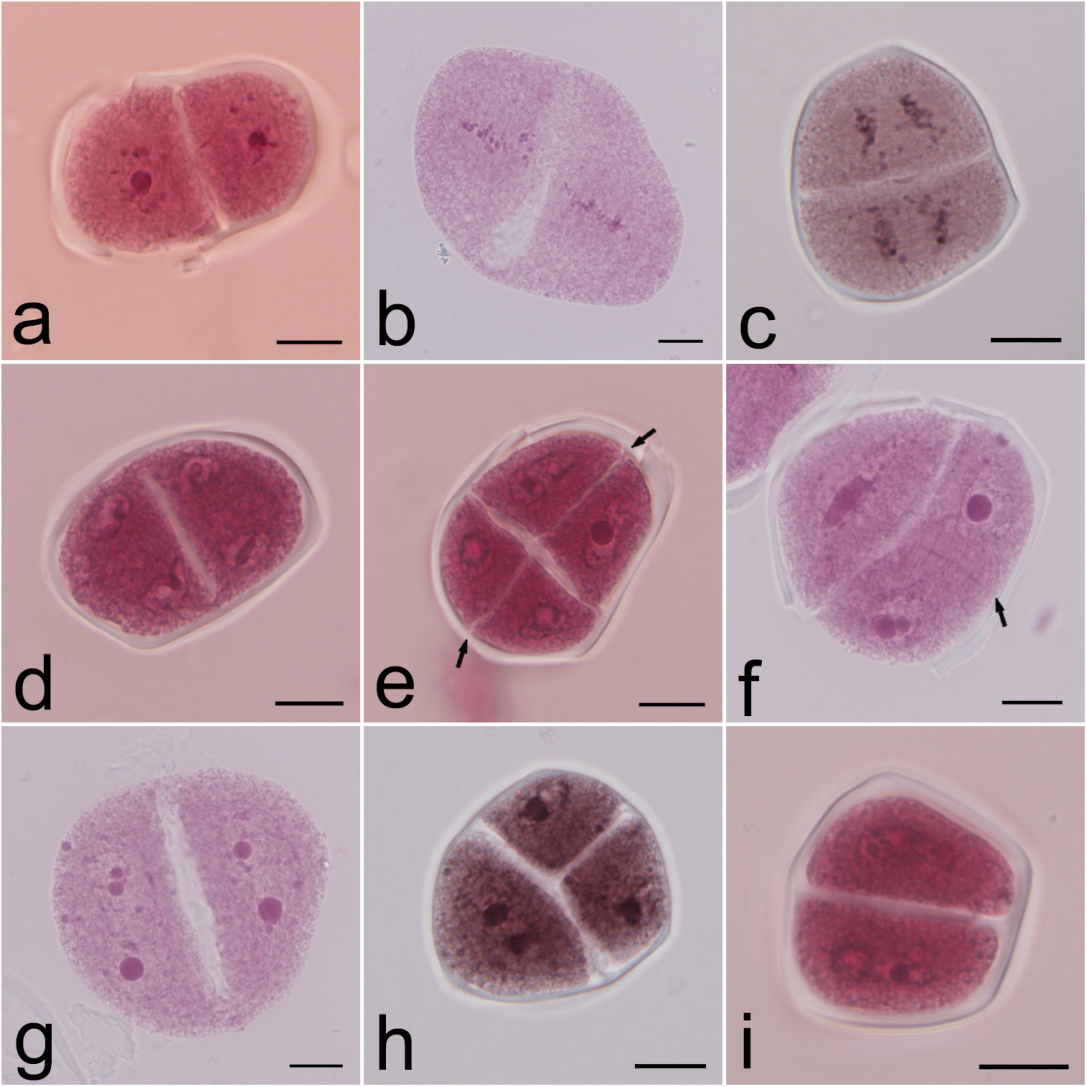
Precocious cytokinesis and nuclear fusion. a–d. Precocious cytokinesis in prophase II (a), metaphase II (b), anaphase II (c) and telophase II (d); e. Completion of successive cytokinesis showing formation of secondary cell plates between sister nuclei (arrows); f. Telophase II with precocious cytokinesis showing a fused nucleus in a daughter cell and separate nuclei by phragmoplast (arrow) in the other cell; g. Telophase II with precocious cytokinesis showing nuclear fusion in both daughter cells; h. Triad with a fused nucleus; i. Dyad with two fused nuclei. Bars are equal to 10 μm.

Precocious cytokinesis was observed in some microsporocytes after the first meiotic division because the cell plate formed in advance to separate the two daughter nuclei into different cytoplasm domains (Fig 3A). During the second meiotic division, the chromosomes in different domains could be positioned in the equatorial region and separated to two different poles to form daughter nuclei (Fig 3B–3D). The frequency of precocious cytokinesis increased from 21.03% at prophase II to 37.12% at telophase II, suggesting that precocious cytokinesis also occurred during the second meiotic division. In some cells with precocious cytokinesis, secondary cell plates formed between sister nuclei to achieve second cytokinesis at telophase II (Fig 3E). The formation of phragmoplasts between sister nuclei defined the position of the cell plate and predicted the occurrence of cytokinesis (Fig 3F). However, fusion of sister nuclei located in the same cytoplasm domain was also observed in some microsporocytes when secondary cell plates failed to form (Fig 3F and 3G), resulting in the formation of dyads and triads in meiotic products (Fig 3H and 3I).

**Fig 4:**
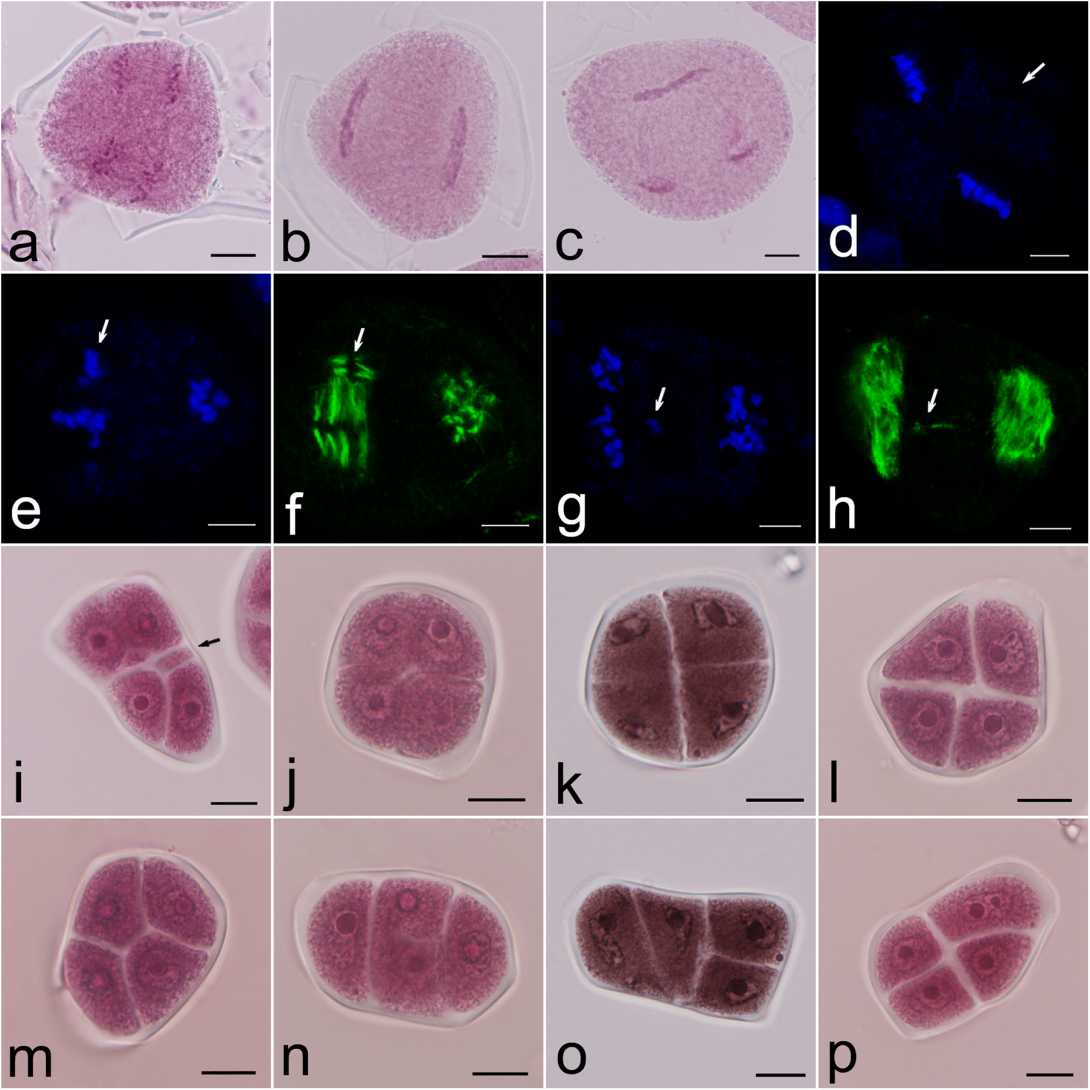
Spindle misorientations and meiotic products. a–c. Anaphase II with parallel spindles (a), fused spindles (b) and tripolar spindles (c); d. Metaphase II with an organelle band (arrow) between two spindles; e–f. Metaphase II with a mini-spindle showing chromosome arrangement (e) and microtubule distribution (f); g–h. Anaphase II with a mini-spindle showing chromosome arrangement (g) and microtubule distribution (h); i. Polyad with microcytes (arrow); j–o. Tetrads with different arrangements of daughter cells; p. Tetrad with unbalanced cytokinesis. Bars are equal to 10 μm in (a)–(c), (i)–(p); bars are equal to 5 μm in (d)–(h).

In some microsporocytes without precocious cytokinesis during the second meiotic division, misoriented spindles, including parallel, fused, and tripolar spindles, were observed (Fig 4A–4C). Fluorescence location analysis showed that there was an organelle layer positioned between two spindles of parallel type (Fig 4D), which separated the spindle domains. In metaphase II, the micronucleus formed isolated chromosomes located beyond the primary chromosome sets (Fig 4E and 4G). The isolated chromosomes led to the formation of mini-spindles in the cytoplasm (Fig 4F and 4H), which gave rise to microcytes in the meiotic products (Fig 4I). The chromosomes in microcytes were eliminated from daughter nuclei.

Among the meiotic products, 1.96% dyads, 3.67% triads and 7.95% polyads were recorded. The meiotic index was 86.42%. Tetrads had different arrangement patterns such as tetrahedral, tetragonal, rhombic, linear, and T-type arrangements (Fig 4J–4P). In some microspores, micronuclei and the primary nucleus were allocated into one microspore (Fig 4O and 4P), which resulted in unbalanced cytokinesis and chromosome segregation.

### Size variation and viability of pollen grains

**Fig 5:**
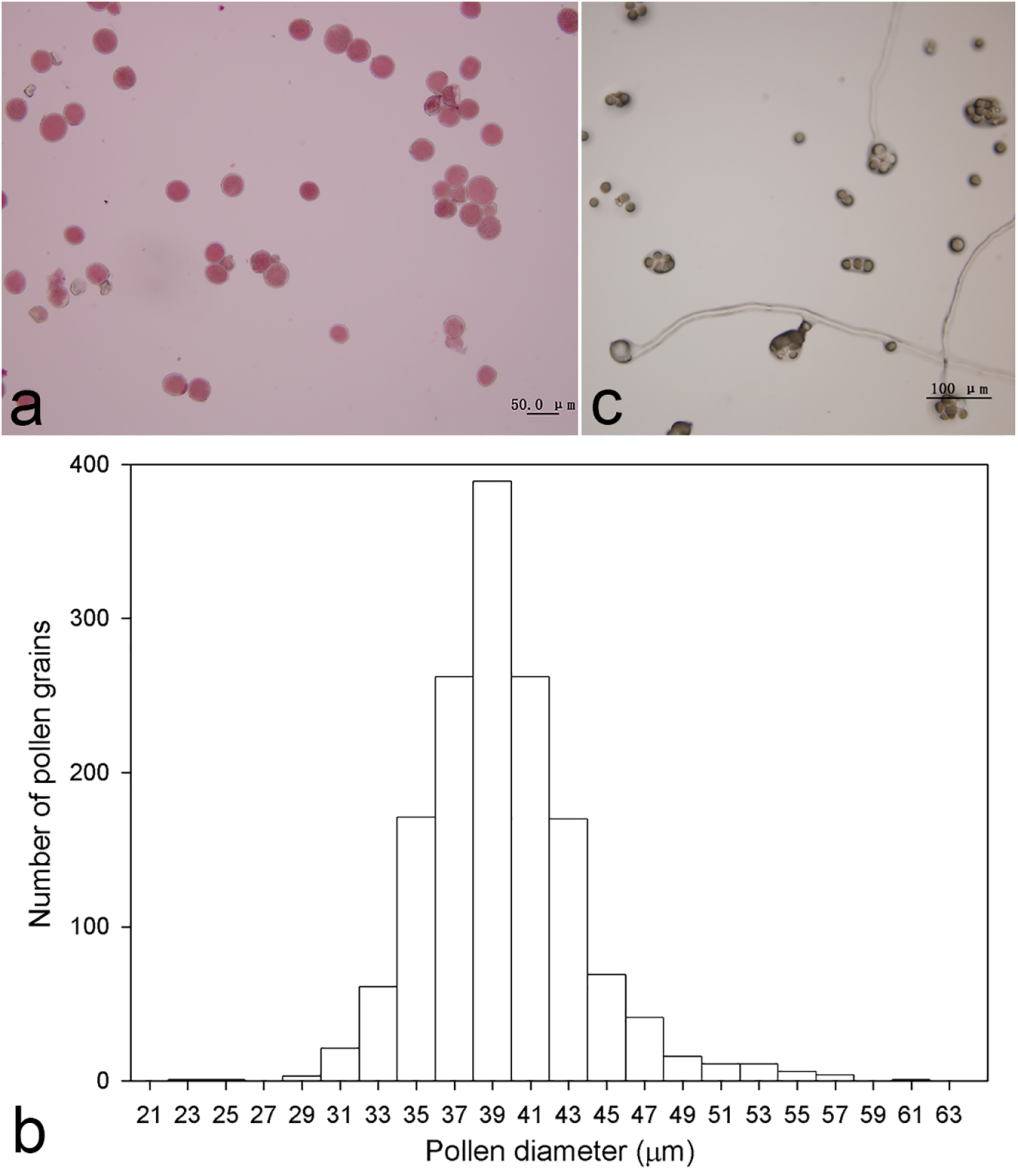
Pollen grains of *P*. *alba* ×*P*. *berolinensis* ‘Yinzhong’ and their germination. a. Pollen morphology of ‘Yinzhong’; b. Histogram of frequency distribution of pollen diameter; c. Pollen germination *in vitro*.

In newly collected pollen samples, approximately 11.3% of the pollen grains were empty and shrunken (Fig 5A). The diameter of the stainable pollen grains ranged from 23.9 to 61.3 μm with an average of 39.6 μm (±4.2). The pollen diameters approximately followed a Gaussian frequency distribution (Fig 5B), suggesting that most ‘Yinzhong’ pollen grains are aneuploid owing to completion of triploid meiosis, although a few large pollen grains may be unreduced. A germination test showed that just 2.78% (±0.38) of pollen grains germinated for this allotriploid, indicating that the viability of ‘Yinzhong’ pollen is poor. The tubes of the germinated pollen grains underwent normal growth on medium (Fig 5C).

### Segregation of ploidy levels among progeny

Fresh pollen from the allotriploid ‘Yinzhong’ was used to pollinate a highly fertile TB03 female parent. Only 691 seeds were collected from 22 catkins, indicating the low pollen fertility of the allotriploid. After seed sowing, 99 seedlings were obtained.

**Fig 6:**
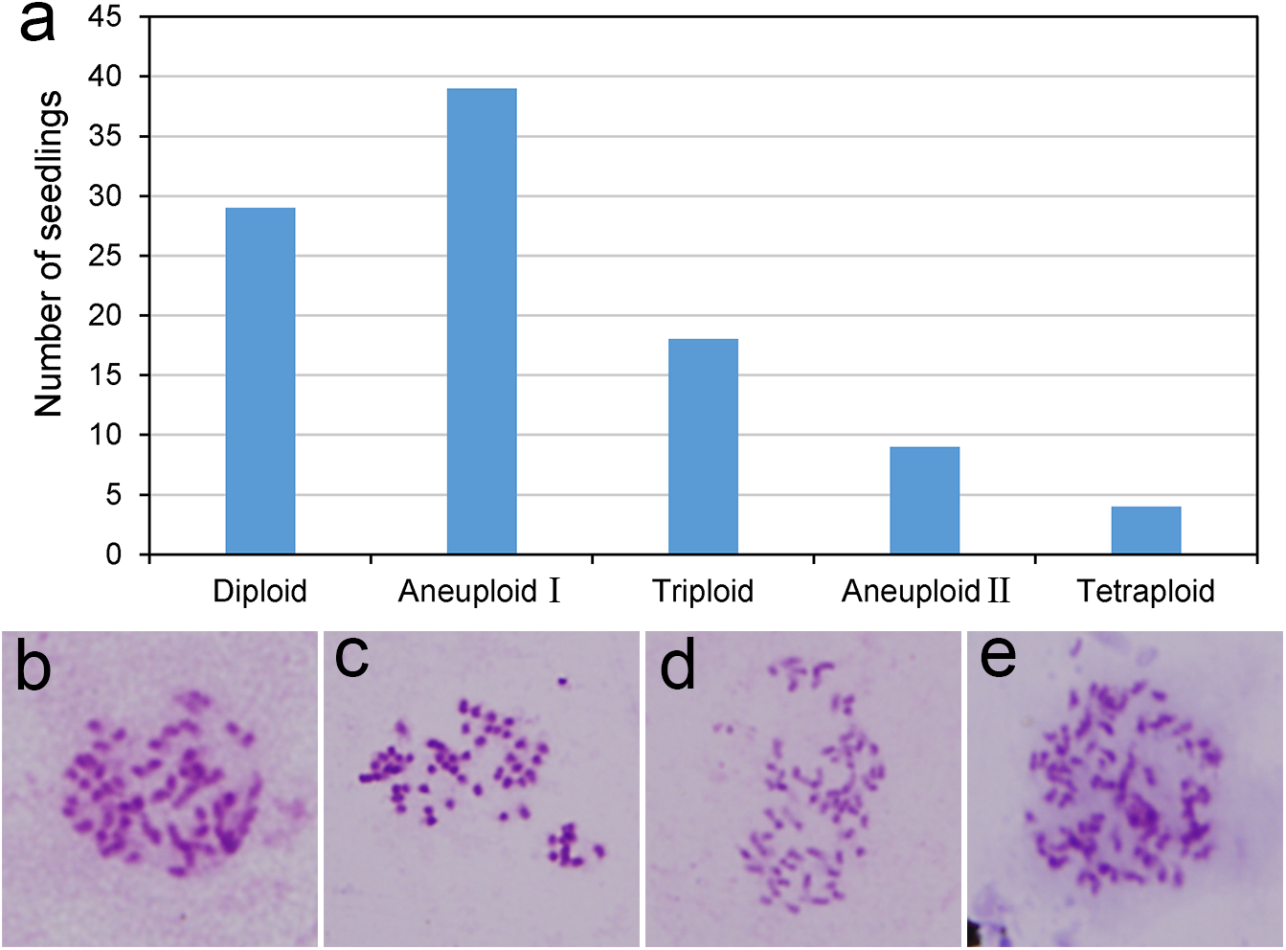
Segregation of ploidy levels in the progeny between *P*. *tomentosa* × *P*. *bolleana* YB03 and *P*. *alba* × *P*. *berolinensis* ‘Yinzhong’. a. Histogram of seedling number with different ploidy levels among the progeny (Aneuploid I: containing chromosome number between 38 and 57; Aneuploid II: containing chromosome number between 57 and 76); b–e. Somatic chromosome counting in some aneuploid individuals. The chromosome numbers are 45 (b), 52 (c), 53 (d), and 68 (e).

The progeny exhibited extensive segregation of ploidy levels, including 29 diploids, 18 triploids, 4 tetraploids and 48 aneuploids (Fig 6A), suggesting that some aneuploid pollen grains of ‘Yinzhong’ are fertile and that unreduced pollen took part in the fertilization to produce seeds. Based on chromosome number, 39 of the aneuploids were classified into “aneuploid I” (Fig 6B–6D) with a chromosome number between 38 and 57, and 9 aneuploids were classified into “aneuploid II” (Fig 6E) with a chromosome number between 57 and 76.

## Discussion

Meiosis in polyploids is characterized by complex chromosomal pairing and chromosome segregation. Imbalanced chromosome pairing and segregation result in the production of lagging chromosomes and micronuclei [11,47]. In an allotriploid white poplar, multivalents, lagging chromosomes and micronuclei were recorded at high frequencies [44]. In this study, the allotriploid ‘Yinzhong’ showed partial failure of uniparental chromosomal pairing, with an average meiotic configuration of 15.2I + 14.0II + 4.6III. Meiotic abnormalites such as precocious chromosome migration, lagging chromosomes, and micronuclei, were also found with high frequency in the allotriploid ‘Yinzhong’, indicating that intersectional hybridization and polyploidization may induce abnormal chromosome segregation in *Populus*. Moreover, chromosome bridges were also observed during meiosis in this study, suggesting that chromosome structural variation occurred in ‘Yinzhong’.

Microsporogenesis of *P*. *alba* occurs normally with the sole exception of several laggards in anaphase I and II [48]. In *P*. *berolinensis*, which is a putative natural hybrid between *P*. *laurifolia* and *P*. *nigra* var. *italica*, abnormal meiotic phenomena such as multinuclei, multinucleoli, monads, dyads, and polyads have been observed [49]. Previous studies on diploid *Populus* hybrids also showed that hybridization tends to induce occurrence of meiotic abnormalities [45,50]. However, compared with the diploid species and hybrids of *Populus*, although meiosis in ‘Yinzhong’ showed similar abnormalities, the frequency of abnormalities was high in ‘Yinzhong’, suggesting that more abnormalities might be induced by its distant genomic composition and polyploidization.

Production of unreduced gametes has been reported in many species and hybrids of the genus *Populus* [44,45,50–54]. There are multiple cytological mechanisms of unreduced gamete formation, which have been reviewed frequently [55,56]. In diploid species of *Populus*, formation of unreduced gametes have been attributed to misorientation of spindles and failure of cytokinesis [52–54], while Wang et al. [44] found that fused and tripolar spindles in the second meiotic division and aberrant cytokinesis result from complete or partial lack of nuclear-based radial microtubule systems (RMSs), leading to unreduced pollen formation in an allotriploid white poplar. In this study, besides fused and tripolar spindles, fusion of sister nuclei within the same cytoplasm domain in microsporocytes with precocious cytokinesis also contributed to nucleus restitution. The fused and tripolar spindles initiated first meiotic division restitution (FDR), and the fusion of sister nuclei induced second meiotic division restitution (SDR). For diploid species, both FDR- and SDR-type unreduced gametes possess complete chromosome sets. For triploids, however, SDR-type gametes are likely to contain incomplete chromosome sets due to unbalanced segregation of homologous chromosomes during the first meiotic division. Therefore, these FDR-type unreduced 3x pollen grains probably resulted in tetraploid production in the progeny, with the triploid functioning as an intermediate in the transition from diploid to tetraploid [18,21].

Occurrence of parallel spindles does not necessarily lead to unreduced gamete formation in some species, including *P*. *tomentosa* [11,54]. During the second meiotic division, organelles, including mitochondria and plastid, are usually located between the two spindles to form a layer, separating the cytoplasm into dyad domains [57]. In this study, an organelle layer was observed in microsporocytes with normal and parallel spindle orientations, which likely prevented the fusion of spindles [58].

Generally, gametes of triploid plants are expected to be highly sterile because most are aneuploid with an incomplete or unbalanced chromosome composition [59]. However, in previous studies on blueberry [13], melon [14], *Musa* [15], apple [16], and *Populus* [12], aneuploid progeny were produced by crossing triploids with diploids, suggesting that some aneuploid gametes may be fertile. In the allotriploid studied here, the wide range of pollen diameter may represent the various chromosome numbers of pollen grains. Nevertheless, the germination test showed that the viability of the pollen grains was low, indicating that some special chromosomal combinations are more likely to be fertile than others [60].

Crossing with polyploids is an effective way to induce variation in ploidy levels. In *Actinidia chinensis* Planch., a cross between a hexaploid and a diploid produced hybrids with extensive ploidy level segregation, including triploids, tetraploids, pentaploids, heptaploids, and octaploids [61]. In aspen, pollen grains from triploid plants have been used to pollinate diploids, resulting in the production of triploids, tetraploids, and aneuploids [12]. In this study, interploidy hybridization between the diploid TB03 and the allotriploid ‘Yinzhong’ also resulted in segregation of ploidy levels among progeny, with diploids, triploids, tetraploids and aneuploids bing produced. This diversity in ploidy levels can be used in polyploidy breeding programs and genetic research into *Populus*.

In the evolution of polyploid plants, compensated aneuploids can be synthesized such that the loss of one chromosome is compensated for by the gain of a chromosome from another type despite having a “euploid” chromosome number [62–64]. Such compensated aneuploids play important roles in maintaining genome balance and chromosome stability [65,66]. In this study, 18 triploids were produced. Although these triploids had the “euploid” 57 chromosomes, they were unlikely to contain three integrated chromosome sets owing to unbalanced segregation of meiotic chromosomes in triploids. It is inferred that these triploids are likely compensated aneuploids.

Aneuploids are an important bridge for chromosomal introgression and substitution in crop breeding. Aneuploids have been systematically studied in wheat to reveal the origin and function of different chromosomes and to locate important genes on chromosomes [20]. Furthermore, chromosome engineering based on aneuploids has produced a series of wheat-alien chromosome addition or substitution lines [67]. However, chromosomal introgression and substitution do not occur in trees owing to a lack of aneuploidy series. In this study, *Populus* aneuploids were produced by crossing with the allotriploid ‘Yinzhong’. Aneuploids are valuable for analysis of chromosomal function and chromosome engineering breeding in *Populus*; thus, it is important to clarify the chromosomal composition of each aneuploid in the progeny.

Karyotypic analysis based on chromosome morphology is difficult for poplar trees because their chromosomes are all small, with the exception of chromosome I. Multicolor fluorescent *in situ* hybridization (McFISH) is a feasible method for analyzing the chromosomal karyotype [68]. Hu et al. [69] located ribosomal DNA and telomere repeat sequence probes in *Populus* chromosomes using McFISH; however, the development of chromosome-specific probes is necessary to identify each chromosome of the *Populus* species. With ongoing advances in molecular biology, array comparative genome hybridization (aCGH), single nucleotide polymorphism (SNP) genotyping, quantitative fluorescent PCR (QF-PCR), and genome sequencing have been used to conduct karyotyping [70–73]. Henry et al. [74] developed a system of dosage-based functional genomics to analyze variations in chromosomal number and structure in poplar, and they precisely detected abundant deletions and insertions of chromosomal segments in poplar progeny derived from crossing with gamma-irradiated pollen. These molecular karyotyping techniques can also be used to investigate features of the chromosomal composition of the aneuploids in this investigation.
